# Impact of continuing medical education in cancer diagnosis on GP knowledge, attitude and readiness to investigate – a before-after study

**DOI:** 10.1186/s12875-016-0496-x

**Published:** 2016-07-26

**Authors:** Berit Skjødeberg Toftegaard, Flemming Bro, Alina Zalounina Falborg, Peter Vedsted

**Affiliations:** 1 Research Unit for General Practice, Department of Public Health, Aarhus University, Bartholins Allé 2, DK-8000 Aarhus, Denmark; 2 Research Centre for Cancer Diagnosis in Primary Care (CaP), Department of Public Health, Aarhus University, Bartholins Allé 2, DK-8000 Aarhus, Denmark; 3 Section for General Medical Practice, Department of Public Health, Aarhus University, Bartholins Allé 2, DK-8000 Aarhus, Denmark

**Keywords:** Denmark, General practice, Continuing medical education, Diagnosis, Early detection of cancer, Knowledge, Attitude, Readiness to investigate, Risk assessment

## Abstract

**Background:**

Continuing medical education (CME) in earlier cancer diagnosis was launched in Denmark in 2012 as part of the Danish National Cancer Plan. The CME programme was introduced to improve the recognition among general practitioners (GPs) of symptoms suggestive of cancer and improve the selection of patients requiring urgent investigation. This study aims to explore the effect of CME on GP knowledge about cancer diagnosis, attitude towards own role in cancer detection, self-assessed readiness to investigate and cancer risk assessment of urgently referred patients.

**Methods:**

We conducted a before-after study in the Central Denmark Region including 831 GPs assigned to one of eight geographical clusters. All GPs were invited to participate in the CME at three-week intervals between clusters. A questionnaire focusing on knowledge, attitude and clinical vignettes was sent to each GP one month before and seven months after the CME. The GPs were also asked to assess the risk of cancer in patients urgently referred to a fast-track cancer pathway during an eight-month period. CME-participating GPs were compared with reference (non-participating) GPs by analysing before-after differences.

**Results:**

One quarter of all GPs participated in the CME. 202 GPs (24.3 %) completed both the baseline and the follow-up questionnaires. 532 GPs (64.0 %) assessed the risk of cancer before the CME and 524 GPs (63.1 %) assessed the risk of cancer after the CME in urgently referred consecutive patients. Compared to the reference group, CME-participating GPs statistically significantly improved their understanding of a rational probability of diagnosing cancer among patients urgently referred for suspected cancer, increased their knowledge of cancer likelihood in a 50-year-old referred patient and lowered the assessed risk of cancer in urgently referred patients.

**Conclusions:**

The standardised CME lowered the GP-assessed cancer risk of urgently referred patients, whereas the effect on knowledge about cancer diagnosis and attitude towards own role in cancer detection was limited. No effect was found on the GPs’ readiness to investigate. CME may be effective for optimising the interpretation of cancer symptoms and thereby improve the selection of patients for urgent cancer referral.

**Trial registration:**

NCT02069470 on ClinicalTrials.gov. Retrospectively registered, 1/29/2014.

**Electronic supplementary material:**

The online version of this article (doi:10.1186/s12875-016-0496-x) contains supplementary material, which is available to authorized users.

## Background

Increasing evidence indicates that timely detection can improve cancer survival [1–3]. Urgent referral to cancer investigation was established in Denmark in 2008 [4], but successful effect of such initiative depends on the patient referral patterns in general practice. As 85 % of cancer patients initially present symptoms to their general practitioner (GP) [3, 5], timely referral may be improved by optimising the GPs’ recognition and interpretation of symptoms as this might lead to earlier diagnosis of cancer. A major challenge for the GP is that most of the symptoms which could signal cancer have a benign cause [6]. Each time such a patient is seen in consultation, the GP must evaluate the risk of cancer, consider the need for investigations and assess the degree of urgency [7]. Therefore, the GP operates under an integrated risk model which considers both basis risk (e.g. patient age, gender and family history) and added risk (e.g. presence or absence of symptoms, clinical findings and test results) [8]. The latest Danish national cancer plan, which was adopted in 2012, launched a continuing medical education (CME) programme in earlier cancer diagnosis to support and further develop the GPs’ decision-making strategies for referral. A specific CME intervention was developed [9] and implemented based on the available evidence [10–12]. The CME focused on several issues, including the diagnostic process in general practice, symptom risk assessment tools (RATs) [13, 14] and risk of false reassurance when interpreting the results of chest X-rays [15, 16] and gynaecological examinations [14].

Enhanced knowledge may provide improved understanding of diagnosing cancer, which may change the GP’s attitude towards own role in cancer detection. Still, attitudes and readiness to investigate are also influenced by the cultural settings and the organisational system in which the GP operates as a gatekeeper to secondary care [17, 18]. However, to our knowledge, no former studies have measured the effect of a specific CME on knowledge, attitude and intentions in the same study, with the same GP participants and intervention.

The aim of this study was 1) to analyse the effect of a standardised CME in earlier cancer diagnosis on the GPs’ knowledge about cancer diagnosis and 2) to assess the GPs’ attitudes towards own role in cancer detection. We also intended 3) to analyse whether the CME changed the GPs’ self-assessed readiness to investigate hypothetical symptomatic patients and 4) to assess the impact of the CME on the GPs’ cancer risk assessment of patients urgently referred for cancer suspicion.

## Methods

### Setting

The study was performed in one of five Danish regions, the Central Denmark Region, with 1.27 million inhabitants, 417 general practices and 831 GPs. In Denmark, GPs own their own practice and operate under a publicly funded mixed remuneration scheme based on a collective agreement with the Danish regions [19]. All GPs have a contract with the public health insurance, which ensures more than 90 % of their earnings. Part of the agreement includes remuneration for participation in CME. Most citizens (98 %) are listed with a specific general practice, and the GP must first be consulted if a citizen needs (free) medical advice, except for emergencies. Therefore, GPs act as gatekeepers to hospitals and private specialists.

### Intervention: continuing medical education

The planning of the CME was initiated by a multidisciplinary team. A working group was established to operationalise the CME, which has been described elsewhere [9]. The CME consisted of a 3-h session with a multifaceted teaching approach [12]. The key aim of the CME was to optimise cancer-related referrals from general practice to hospitals by reducing the GPs’ referral threshold and to increase their knowledge about cancer symptoms in an attempt to identify underlying cancers at an earlier stage. Barriers on GP-level were addressed in a reflective debate among the GPs [9]. All GPs were provided with the available risk assessment charts on lung, colorectal, ovarian and prostate cancer [13, 14]. However, the CME focused on earlier detection of all cancer types. The CME was conducted by a team of six persons with different health-care backgrounds [9].

### Overall study design and recruitment of GPs

To increase CME attendance, geographical accessibility was ensured by conducting eight local meetings. All GPs in the region were assigned to one of eight clusters based on geography. All GP clusters were invited to the CME in the period of September 2012 to May 2013 with 3-week intervals. The Regional Cancer Quality Unit decided the random order in which the clusters were offered the CME intervention. The CME was announced by mail invitations two months and again one month before the CME was planned to take place. Each GP was registered upon arrival at the CME [9]. We performed a before-after study embedded in a stepped-wedge enrolment of the CME intervention.

#### Questionnaire data

GPs who had already responded to a similar questionnaire (International Cancer Benchmarking Partnership study, module-3 (ICBP-M3)) [20] in September 2012 were excluded, and 751 GPs were invited to complete the baseline questionnaire. The GPs received an e-mail invitation with a link to the online survey one month before their CME was scheduled to take place. GPs who responded to the baseline survey were invited to complete the follow-up questionnaire seven months after their CME date. Reminders were sent to non-respondents 14 days after the primary invitation in both surveys. Remuneration was 33 euros for completing the baseline questionnaire and 17 euros for completing the follow-up questionnaire.

The online questionnaire consisted of single items and vignettes. The items were developed based on the elements included in the CME to measure the CME effect on knowledge about cancer diagnosis (6 items), attitude towards own role in cancer detection (7 items) and attitude towards practical aspects of urgent referral [9]. The vignettes were validated and tested in the ICBP-M3 project [20]. They measured readiness to investigate when a hypothetical patient presented with symptoms suggestive of lung cancer (two vignettes), colorectal cancer (two vignettes) or ovarian cancer (one vignette). The English versions of the vignettes were translated into Danish [9].

### GP knowledge

GP knowledge about cancer diagnoses comprised 1) cancer as a condition with a low prevalence (one item), 2) positive predictive values (PPVs) for cancer of selected symptoms (two items), 3) diagnostic pitfalls (two items) and 4) initial cancer presentation (one item). The wording of each item initiated with “what is the likelihood that…” or ‘what is the proportion of…” and the GP was requested to state the response as a percentage (0–100 %). Based on the available evidence, the research group agreed on the most appropriate response (MAR) test area as a percentage range for each of the six items (Additional file 1).

### GP attitude

The GP’s attitude towards own role in cancer detection, which is related to the GP’s interpretation of the balance between benefits and harms of early diagnostic testing, was measured by statements addressing the concerns about 1) overusing healthcare services or distressing the patient when an urgent referral does not result in a cancer diagnosis (two items), 2) risking over-diagnosis when ordering tests (one item), 3) communicating cancer risk with patient and fast-track coordinator (two items) and 4) exploring the GP’s referral threshold for urgent cancer investigation (two items). The GP’s attitude towards practical aspects of urgent referral was measured by statements addressing the GP’s experience with 1) use of urgent referral (complicated and/or time-consuming), 2) delivery of written patient instructions and 3) use of information from the online cancer guideline. Responses were given on a 5-point Likert scale. The research group agreed to dichotomise the responses for each attitude item based on the two most appropriate responses (i.e. agree/strongly agree) versus the remaining three response options including neutral.

### GP readiness to investigate

The GPs were presented with two randomly chosen vignettes at baseline and another two vignettes at follow-up. Each vignette included a description of a patient’s initial symptom presentation and asked the GP to decide which action to take. If the GP chose an expectant strategy, the case developed further and the patient returned with new and more severe symptoms [20] (Additional file 2).

### GP cancer risk assessment of urgently referred patients

All 831 GPs in the Central Denmark Region received a pad with 25 one-page forms. The GPs were requested to complete a form each time a patient was urgently referred for suspected cancer [9, 21] during an 8-month period (1 September 2012–1 May 2013). Patients investigated in a fast-track pathway were also identified in the patient administrative system for hospital care. If the GP form was missing for a patient identified in the register, the GP received a form for that particular patient and was requested to complete it.

The form included information about the patient’s personal identification number, the date of referral and the GP’s assessment of the patient’s risk of cancer (0–100 %) at the time of the referral [9]. Remuneration was 100 euros for completion of forms during the 8-month period. The form was processed using Teleform Version 8.0 (Cardiff Software Inc., San Marcos, CA, USA). Handwritten data were coded by the first author (BST) before scanning [22]. All optically scanned data were checked for outliers and discrepancies.

## Analyses

GPs were divided into a CME-participating group and a non-participating reference group. The time point for a CME-session was identical with the point for crossover from before to after, and thus it constituted a step in the before-after models. Data were collected before and after the CME to provide paired responses for each outcome variable. GP characteristics were described for the entire study base, for the GPs who completed baseline and follow-up surveys and for GPs who assessed cancer risk in referred patients.

Responses to attitude and knowledge items were presented as proportions of the GPs who responded most appropriately (Additional File 1 for knowledge items). Attitude and knowledge levels before and after CME were compared within and between groups by conditional fixed-effects Poisson regression based on cluster-robust standard errors (SEs). Within-individual differences were used to estimate effects, and each GP was used as own control. We used the robust SEs to relax the assumption behind the Poisson model that the variance was equal to the mean [23]. The SEs were corrected for clustering at step level. Effects within groups were reported as risk ratios (RRs). Comparison of effects between groups was reported as a ratio of RRs.

GP readiness to investigate was analysed through the proportions of vignettes where the GP made a definitive action after first and after second phase. Because of the binary data format, comparisons of responses before and after CME were performed by using multilevel mixed-effects logistic regression to ensure that within-GP correlations were taken into account [24]. The model allowed for random effects at GP level. The time of observation (before and after CME) was treated as a fixed-effect variable. The effects within groups were reported as odds ratios (ORs). Comparison of effects between groups was reported as a ratio of the ORs. The analyses were adjusted for study step and type of vignette.

GP cancer risk assessment of urgently referred patients was described as mean risk with 95 % confidence interval (CI) and median with interquartile interval (IQI), as the population was not normally distributed. Comparisons of risk assessment before and after CME within and between groups were performed using multilevel mixed-effects linear regression. The model was fitted to take into account within-GP correlations and GP clustering in clinics. The time of observation (before and after CME) was treated as a fixed effect variable. To satisfy the assumptions of the model and be able to compare mean risks, the dependent variable was square root transformed. The analyses were adjusted for study step, patient gender and patient age.

All statistical analyses were performed using Stata software version 13.0 (StataCorp LP, College Station, TX, USA).

## Results

### Study population

**Fig. 1:**
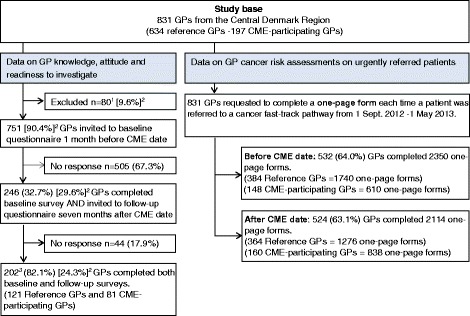
Flowchart of the data collection. The left part illustrates the GP completion of questionnaires at baseline (1 month before the CME) and at follow-up (7 months after the CME). The right part illustrates the consecutive GP completion of one-page forms, including assessed risk of cancer in referred patients before and after the CME. ^1^GPs who completed a similar questionnaire in the ICBP study, module 3, September 2012. ^2^Proportion of study base. ^3^196 GPs completed both baseline and follow-up vignettes (116 reference GPs and 80 CME-participating GPs). 6 GPs were lost to follow-up due to a technical failure of the online survey system

The study base consisted of 831 GPs (Table 1). A total of 197 GPs (23.7 %) participated in the CME; they did not differ from the study base in age, but the CME-participating GPs were less likely to be male and solo GPs. Baseline and follow-up questionnaires were completed by 202 GPs (24.3 %). Of these, the CME-participating GPs were younger, less likely to be male and solo GPs, whereas reference GPs were younger, less likely to be female, but they did not differ in clinic type from the study base (Table 1). Due to technical difficulties with the online survey, 6 GPs were not able to complete both baseline and follow-up vignettes and were excluded from the vignette analyses (Fig. 1).

**Table 1:** Characteristics of GPs. Age, gender and type of clinic are shown for all GPs in the Central Denmark Region (study base), GPs responding to the knowledge and attitude questionnaire, GPs completing the vignettes and GPs assessing risk of cancer for continuous patients. GPs are divided into the CME-participating GPs and the reference GPs

		GP characteristics		
	*N* (%)	Mean age Years (95 % CI)	Proportion of males % (95 % CI)	Proportion of solo GPs % (95 % CI)
Study base				
Total	831 (100)	52.3 (51.7; 52.8)	54.3 (50.9; 57.7)	24.1 (21.2; 27.0)
Reference GPs	634 (76.3)	52.4 (51.7; 53.0)	56.5 (52.6; 60.3)	26.0 (22.6; 29.4)
CME-participating GPs	197 (23.7)	51.9 (50.8; 53.0)	47.2 (40.2; 54.2)	17.8 (12.4; 23.2)
GPs that completed knowledge and attitudes items				
Reference GPs	121 (19.1)	49.8 (48.3; 51.4)	57.9 (48.9; 66.8)	24.0 (16.3; 31.7)
CME-participating GPs	81 (41.1)	50.7 (48.8; 52.5)	46.9 (35.8; 58.0)	18.5 (9.9; 27.2)
GPs that completed vignettes				
Reference GPs	116 (18.3)	49.9 (48.4; 51.5)	58.6 (49.5; 67.7)	23.3 (15.5; 31.1)
CME-participating GPs	80 (40.6)	50.5 (48.7; 52.4)	47.5 (36.3; 58.7)	18.8 (10.0; 27.5)
GPs that assessed risk of cancer on urgently referred patients				
Reference GPs, before	384 (60.6)	51.6 (50.8; 52.4)	52.6 (47.6; 57.6)	26.8 (22.4; 31.3)
Reference GPs, after	364 (57.4)	51.7 (50.8; 52.6)	53.6 (48.4; 58.7)	22.3 (18.0; 26.5)
CME-participating GPs, before	148 (75.1)	51.6 (50.3; 52.9)	45.9 (37.8; 54.1)	17.6 (11.4; 23.8)
CME-participating GPs, after	160 (81.2)	51.9 (50.6; 53.1)	46.9 (39.1; 54.7)	15.6 (9.9; 21.3)

Cancer risk assessment of urgently referred patients was completed by 532 GPs (64.0 %) before the CME and 524 GPs (63.1 %) after the CME. The CME-participating GPs were less likely to be male and solo GPs, but did not differ in age, whereas the reference GPs did not differ in age, gender and clinic type compared to the study base (Table 1).

### CME effect on GP attitude

The CME-participating GPs showed a statistically significant appropriate change in 2 of 7 items addressing the attitude towards own role in cancer detection compared to the reference GPs (Table 2). After the CME, the participating GPs were more likely to disagree with the following two statements: ‘If a patient referred by me to a cancer fast-track pathway turns out not to have cancer, unnecessary strain has been placed on the patient’ and ‘The more of the patients referred by me to a cancer fast-track pathway turn out to have cancer, the better a doctor I am’*.*

**Table 2:** The CME effect on GPs’ attitude towards own role in cancer detection. The proportion of GPs responding most appropriately is shown for each of the items; one month before (Before) and seven months after (After) the CME. GPs are divided into two groups: CME-participating group and reference group. An effect within a group is shown as a risk ratio (RR0 for the Reference group; RR1 for the CME-participating group). Comparisons between groups are shown as ratio of risk ratios (RR1/RR0)

		Reference group			CME-Participating group			Comparison between groups
		*N* = 121			*N* = 81			
	Most appropriate answers	Before	After	Before vs. after	Before	After	Before vs. after	RR1/RR0 (p*)
		% (n)	% (n)	RR0 (p*)	% (n)	% (n)	RR1 (p*)	
If a patient referred by me to a cancer fast-track pathway turns out not to have cancer, it is overuse of health services.	*Strongly disagree - disagree*	86.8 (105)	84.3 (102)	0.97 (0.419)	81.5 (66)	87.7 (71)	1.08 (0.163)	1.11 (0.241)
If a patient referred by me to a cancer fast-track pathway turns out not to have cancer, unnecessary strain has been placed on the patient.	*Strongly disagree - disagree*	83.5 (101)	79.3 (96)	0.95 (0.221)	75.3 (61)	79.0 (64)	1.05 (0.275)	1.11 **(<0.001)**
The more of the patients referred by me to a cancer fast-track pathway turn out to have cancer, the better a doctor I am.	*Strongly disagree - disagree*	57.9 (70)	62.0 (75)	1.07 (0.224)	59.3 (48)	79.0 (64)	1.33 (**0.003**)	1.24 **(0.013)**
I am a good doctor when I refer a patient to a cancer fast-track pathway based on a reasonable suspicion, as it quickly clarifies the suspicion.	*Strongly agree- agree*	81.0 (98)	81.8 (99)	1.01 (0.823)	86.4 (70)	82.7 (67)	0.96 (0.397)	0.95 (0.537)
I am reluctant to order tests because of the risk of over-diagnosis.	*Strongly disagree - disagree*	75.2 (91)	75.2 (91)	1.00 (1.000)	71.6 (58)	75.3 (61)	1.05 (0.308)	1.05 (0.314)
I find it hard to mention cancer suspicion to a patient with alarm symptoms of cancer.	*Strongly disagree - disagree*	81.0 (98)	83.5 (101)	1.03 (0.402)	74.1 (60)	75.3 (61)	1.02 (0.704)	1.00 (0.813)
I feel well prepared to communicate with the cancer pathway coordinators.	*Strongly agree- agree*	82.6 (100)	78.5 (95)	0.95 (0.224)	79.0 (64)	77.8 (63)	0.98 (0.806)	1.03 (0.697)

Bold = significance level of p ≤ 0.05. *corrected for clustering at the study-step-level

No differences were found for the 6 items addressing attitude towards practical aspects of urgent referral for suspected cancer (not reported in table).

### CME effect on GP knowledge about cancer diagnosis

The CME-participating GPs showed a statistically significant appropriate change in only 1 out of 6 items addressing GP knowledge about cancer diagnosis compared to the reference GPs. They increased their knowledge regarding the likelihood that a patient aged 50 would have cancer at the time of urgent referral (Table 3). Although not significantly, the CME-participating GPs tended to have increased knowledge compared to the reference GPs on the likelihood that a patient aged 40 and above would have colorectal cancer the first time the patient presented unintended weight loss and newly onset constipation in general practice (Table 3).

**Table 3:** The CME effect on GPs’ knowledge about cancer diagnosis. The proportion of GPs responding most appropriately (MAAA) is shown for each item; one month before (Before) and seven months after (After) the CME. GPs are divided into two groups: CME-participating group and reference group. An effect within a group is shown as a risk ratio (RR0 for the Reference group; RR1 for the CME-participating group). Comparisons between groups are shown as ratio of risk ratios (RR1/RR0)

		Reference group			CME Participating group			Comparison between groups
		*N* = 121			*N* = 81			
	MAAA	Before	After	Before vs. after	Before	After	Before vs. after	RR1/RR0 (p*)
		% (n)	% (n)	RR0 (p*)	% (n)	% (n)	RR1 (p*)	
What is the likelihood that a 50-year-old patient having cancer at the time you choose to refer the patient to a cancer fast-track pathway?	2–10 %	29.8 (36)	38.8 (47)	1.31 **(<0.001)**	23.5 (19)	65.4 (53)	2.79 **(<0.001)**	2.13 **(0.009)**
What is the likelihood that a patient aged 40 years or more, who is smoker, has lung cancer the second time s/he presents with haemoptysis in your practice?	5–20 %	33.1 (40)	34.7 (42)	1.05 (0.698)	33.3 (27)	58.0 (47)	1.74 **(0.010)**	1.63 (0.160)
What is the likelihood that a patient aged 40 years or more has colorectal cancer the first time that s/he presents with unintended weight loss and new onset of constipation in your practice?	2–6 %	8.3 (10)	9.9 (12)	1.19 **(0.039)**	6.2 (5)	28.4 (23)	4.6 **(<0.001)**	3.83 (0.055)
What is the likelihood that a lung cancer cannot be detected on a chest x-ray at the time of diagnosis?	≥15 %	81.8 (99)	86.0 (104)	1.05 (0.741)	76.5 (62)	81.5 (66)	1.06 (0.243)	1.07 (0.309)
What is the proportion of patients with colorectal cancer who presented an alarm symptom as the first sign of the disease to his/her general practitioner?	≤60 %	81.8 (99)	86.0 (104)	1.05 (0.449)	82.7 (67)	90.1 (73)	1.09 **(0.006)**	1.04 (0.614)
What is the proportion of patients with ovarian cancer who can be detected by a pelvic examination (palpation) in general practice at the time of diagnosis?	≤41 %	90.0 (110)	94.2 (114)	1.04 (0.078)	85.2 (69)	92.6 (75)	1.09 **(0.012)**	1.05 (0.322)

Bold = significance level of p ≤ 0.05. *corrected for clustering at the study-step-level

### CME effect on GP readiness to investigate (vignettes)

The proportion of vignettes where GPs made a definitive action at the end of the second phase has been significantly increased within the CME-participating group, but no significant differences in before-after changes in self-assessed readiness to investigate were found between the two groups (Table 4).

**Table 4:** The CME effect on GPs’ self-assessed readiness to investigate. The proportions of vignettes where the GP made a definitive action after first or second phase of the vignette; one month before (Before) and seven months after (After) the CME. GPs are divided into two groups: CME-participating group and reference group. An effect within a group is shown as an odds ratio (OR0 for the Reference group; OR1 for the CME-participating group). Comparisons between groups are shown as a ratio of odds ratios (OR1/OR0)

	Reference group N (vignettes) = 232 N (GPs) = 116			CME-participating group N (vignettes) = 160 N (GPs) = 80			Comparison between groups
	Before	After	Before vs. after	Before	After	Before vs. after	OR1/OR0* (p)
	% (n)	% (n)	OR0* (p)	% (n)	% (n)	OR1* (p)	
Definitive action, at the end of first phase	37.9 (88)	40.1 (93)	1.19 (0.444)	36.9 (59)	43.8 (70)	1.70 (0.055)	1.43 (0.316)
Definitive action, at the end of second phase	77.2 (179)	77.6 (180)	1.04 (0.872)	81.9 (131)	88.8 (142)	**2.02 (0.043)**	1.94 (0.119)

Bold = significance level of *p* < 0.05. *adjusted for a study-step and type of vignette

### CME effect on GP cancer risk assessment of urgently referred patients

The CME-participating GPs statistically significantly lowered their assessed risk of cancer in urgently referred patients compared to the reference GPs (Table 5).

**Table 5:** The CME effect on GP cancer risk assessment when referring consecutive patients for urgent referral. The numbers of assessing GPs and assessed patients are shown together with the median (inter quartile interval (IQI)) and the mean (95 % CI) assessed risk of cancer before and after the CME. GPs are divided into two groups: CME-participating group and reference group. An effect within a group and comparisons between groups are shown as regression coefficients

	Reference group			CME-participating group			Comparison between groups
	*N* = 634			*N* = 197			
	Before	After	Before vs. after	Before	After	Before vs. after	Regression coefficient* (p)
			Regression coefficient* (p)			Regression coefficient * (p)	
GPs (n (%))	384 (60.6)	364 (57.4)		148 (75.1)	160 (81.2)		
Patients (n)	1740	1276		610	838		
Median risk (% (IQI))	30 (10–50)	30 (10–50)		30 (10–50)	15 (10–50)		
Mean risk (% (95 % CI))	39.6 (38.1; 41.0)	38.8 (37.1; 40.4)	−0.17 (0.063)	39.0 (36.6; 41.4)	27.4 (25.6; 29.3)	−1.10 **(<0.001)**	−0.93 **(<0.001)**

Bold = significance level of *p* < 0.05. *adjusted for a study-step and patient’s gender and age, on square root scale

## Discussion

### Main findings

We found that one fourth of the GPs participated in a CME on earlier cancer diagnosis. The standardised CME lowered the GP-assessed cancer risk of urgently referred patients and showed a limited effect on the GPs’ knowledge about cancer diagnosis and attitude towards own role in cancer detection. The CME had no effect on the GPs’ self-assessed readiness to investigate or the GPs’ attitude towards practical aspects of urgent referral.

### Strength and limitations

The before-after design with geographical clusters of GPs receiving CME in a random order three weeks a part made it possible to perform a robust evaluation with correction for baseline measures of a rather naturalistic experiment. Randomisation at an individual GP level was considered unfeasible due to interaction of GPs within practices. Furthermore, it was preferred to invite all GPs from one cluster to the same CME meeting because geographical acceptability had high priority, and two meetings in each cluster area was found unfavourable due to financial and time costs.

A principal strength is the well-defined study population of GPs described by age, gender and clinic type. Other factors of interest could have been the socio-demographic characteristics of the patient population.

The geographical clusters were divided into CME-participating and reference GPs based on the GP’s choice of participation. Spill-over effect from the CME-participating GPs to the reference GPs could have underestimated the actual effect of the CME. Other initiatives could also have influenced the change from baseline to follow-up, although no competitive regional courses were offered in the study period. We attempted to correct for influence of calendar time by including study steps into the modelling of data.

A major strength is the standardised CME with a detailed description of CME elements, training process and context, which will allow primary health care settings elsewhere to use our findings and adapt them to their context.

Data was collected through a questionnaire developed ad hoc to assess the effect of the CME. The questionnaire included single items specifically targeting the content of the CME. This adaptation may be a potential weakness compared with instruments with established measurement properties, but we found it necessary because relevant validated measures were sparse. The questionnaire was developed in consideration of qualitative and quantitative pilot studies [9]. As paired items were analysed before and after the CME, the possible bias from using ad hoc items was diminished.

The vignettes were developed and validated in an international primary care setting (ICBP-M3) [20, 25]. Evidence suggests that using vignettes in a survey correlates well in clinical practice [26]. The GPs were aware that the questionnaires aimed to evaluate a CME in early cancer diagnosis, and their responses might have been different in a blinded study. Such potential information bias may affect both baseline and follow-up responses in each of the two GP groups, but it is difficult to assess in which direction this may have affected the findings.

A limitation of the study is the modest response rate. As a consequence, selection bias may also affect the findings. We found evidence of such selection bias, but the effect is difficult to establish. Another limitation is the low participation in the CME. GPs may generally select educational activities in areas of their interest and thus may already perform well in these areas [27]. Nevertheless, in this study, the reference GPs tended to response more appropriately at baseline, but we found no statistically significantly differences between the two groups when comparing the baseline responses.

Apart from GP knowledge and referral threshold, the GP-assessed cancer risk of an urgently referred patient depends on e.g. suspected cancer type, symptom severity, symptom duration and genetic disposition for cancer in combination with patient’s age and gender. As we included nearly 4,500 urgently referred patients and adjusted for patient age and gender, the lack of adjustment can be neglected due to an equal variation of patients in each of the two GP groups both before and after the CME. The strategy of reminding the GPs of possible urgent referrals every second week on the basis of hospital registers ensured data completeness. Before being reminded, the reference GPs completed 40.0 % before and 38.6 % after the CME of the one-page forms, whereas the CME-participating GPs completed 47.2 % before and 50.2 % after the CME (not reported in the tables). Recall bias may have occurred when GPs were reminded as a later response may mean that the result of the investigation was known. However, the propensities of the prospective data were almost unchanged from before to after within group. Between groups, misclassification may exist as reference GPs responded less prospectively than the CME-participating GPs which may tend to underestimate the CME-effect.

The national strategy to improve early diagnosis of cancer included a lowering of the GPs’ threshold for referring patients for further investigation. Our study indicated such an effect of the CME, which improved the GP-assessed cancer risk of a patient suspected for cancer at the time of referral. As the GPs had high baseline knowledge on diagnostic pitfalls and initial cancer presentation, the ceiling effect made it difficult to demonstrate an effect. Improvements may still be clinically relevant but the ceiling needs to be taken into account when deciding on interventions strategies, including considerations of making such CME activities compulsory.

### Comparison with other studies

To our knowledge, our study is the first to evaluate a comprehensive and primary-care-based intervention on knowledge, attitudes and intended behaviour related to earlier cancer diagnosis, with the same participants and intervention. However, some studies have assessed parts of our intervention. The use of RATs in general practice was evaluated in the UK and was associated with increased diagnostic activity [28]. In Denmark, the use of RAT for lung cancer as part of a CME was associated with increased referral rate to direct chest CT scan [29]. It is unknown whether the effect found in our study may persist over time. Still, the follow-up of 7 months is longer than in many other studies of educational effects [12].

## Conclusion

The CME lowered the GP-assessed cancer risk of urgently referred patients and showed a limited effect on GPs’ knowledge about cancer diagnosis and attitude towards own role in cancer detection; the CME had no effect on self-assessed readiness to investigate and GPs’ attitude towards practical aspects of urgent referral. A standardised CME may be effective in changing both GP attitude and knowledge including risk assessment, which may be drivers for change in the clinical performance.

## Abbreviations

CI, confidence interval; CME, continuing medical education; GP, general practitioner; ICBP-M3, International Cancer Benchmarking Partnership, Module 3; IQI, interquartile interval; MAR, most appropriate response; OR, odds ratio; RAT, risk assessment tool; RR, risk ratio; SE, standard error

## Additional files

Additional file 1: Table S1. Evidence for the most appropriate response, the predefined most appropriate response area and the testing area for each of the knowledge items. (PDF 113 kb) Additional file 2: Table S2. Description of the vignettes. (PDF 92 kb)
