# Supplementary material for: Impact of continuing medical education in cancer diagnosis on GP knowledge, attitude and readiness to investigate – a before-after study
Source: BMC Fam Pract. 2016 Jul 26;17:95. doi: 10.1186/s12875-016-0496-x (PMC4962470; doi:10.1186/s12875-016-0496-x)
Supplement: Additional file 1: Table S1. — Evidence for the most appropriate response, the predefined most appropriate response area and the testing area for each of the knowledge items. (PDF 113 kb) [file 12875_2016_496_MOESM1_ESM.pdf]

Additional File 1 *Evidence for the most appropriate response (MAR), the predefined MAR range and the MAR test area to each of the knowledge items*

Knowledge items	Evidence for most appropriate response	MAR	MAR range	MAR test area
What is the likelihood that a 50-year-old patient having cancer at the time you choose to refer the patient to a cancer fast-track pathway?	**Evidence for cancer symptoms´ PPVs:**<br>**Hamilton W. Br J Cancer 2009**. The CAPER studies: five case-control studies aimed at identifying and quantifying the risk of cancer in symptomatic primary care patients. **3-8%**<br>The actual risk of cancer when the GP refers might be higher than the symptoms PPV since the patient could present more signs. If the PPV for cancer is over 5-10% the patient perhaps should have been referred at an earlier time point.<br>**Evidence for PPV for diagnosing cancer in fast track referrals:**<br>**Meechan et al. Br J Gen Pract. 2012**: Variation in use of the 2-week referral pathway for suspected cancer: a cross-sectional analysis. All cancer types: **9-14%**<br>**Guldbrandt et al. PLoS One 2014**: Implementing Direct Access to Low-Dose Computed Tomography in General Practice—Method, Adaption and Outcome. **10.2%** (Lung cancer)<br>**Evidence for PPV for diagnosing cancer in fast track referral for a 50-year-old patient.**<br>**Baughan. Br J Gen Pract. 2011**: Urgent suspected cancer referrals from general practice: audit of compliance with guidelines and referral outcomes.<br>40-49 years: **8.6%,** 50-59 years: **14.3%**	5%	2-10%	2% ≥R≤ 10%
What is the likelihood that a patient aged 40 years or more, who is smoker, has lung cancer the second time s/he presents with haemoptysis in your practice?	**Evidence for PPV of a repeated cancer symptom for lung cancer:**<br>Hamilton W. MD thesis, 2005: Towards earlier diagnosis of cancer in primary care: a population-based case-control study of colorectal, lung and prostate cancer. **12%**	12%	5-20%	5% ≥R≤20%
What is the likelihood that a patient aged 40 years or more has colorectal cancer the first time that s/he presents with unintended weight loss and new onset of constipation in your practice?	**Evidence for PPV of combined cancer symptoms for colorectal cancer:**<br>Hamilton W. BJC 2009: The CAPER studies: five case-control studies aimed at identifying and quantifying the risk of cancer in symptomatic primary care patients. **3%**	3%	2-6%	2% ≥ R≤ 6%
What is the likelihood that a lung cancer cannot be detected on a chest x-ray at the time of diagnosis?	**Evidence for risk of negative chest X-ray despite lung cancer**<br>Bjerager. PhD thesis: Delay in diagnosis and treatment of lung cancer. **18%**<br>Stapley et al: Negative chest X-rays in primary care patients with lung cancer. Br J Gen Pract. 2006 **23%**<br>Quekel et al. Chest 1999: Miss rate of lung cancer on the chest radiograph in clinical practice. **19%**	20%	15-25%	R≥ 15%
What is the proportion of patients with colorectal cancer who presented an alarm symptom as the first sign of the disease to his/her general practitioner?	**Evidence for initial symptom presentation for cancer patients**<br>Nielsen et al. Ugeskr. Læger [Weekly Journal for Physicians (Danish medical journal)].2010: Symptom presentation in cancer patients in general practice. **49%**	49%	40-60%	R≤ 60%
What is the proportion of patients with ovarian cancer who can be detected by a pelvic examination in general practice at the time of diagnosis?	**Evidence for that pelvic examination is not useful to exclude the risk of ovarian cancer**<br>Hamilton et al. BMJ 2009: Risk of ovarian cancer in women with symptoms in primary care: population based case-control study. **33%**	33%	25-41%	R≤ 41%
