# Supplementary material for: Impact of continuing medical education in cancer diagnosis on GP knowledge, attitude and readiness to investigate – a before-after study
Source: BMC Fam Pract. 2016 Jul 26;17:95. doi: 10.1186/s12875-016-0496-x (PMC4962470; doi:10.1186/s12875-016-0496-x)
Supplement: Additional file 2: Table S2. — Description of the vignettes. (PDF 92 kb) [file 12875_2016_496_MOESM2_ESM.pdf]

Additional file 2 *Description of the vignettes. The history at first and second phase for each of the five vignettes. The options of management are divided into definitive actions and non-definitive actions. Positive predictive values (PPV) are noted for each phase. (Rose et al. BMJ Open. 2015 May 27; 5(5):e007212)*

Vignette	Phase	PPV (%)	Anamneses of hypothetical patients	Definitive actions	Non-definitive actions
1 Lung	1	0.9	68-year-old female, ex-smoker with persistent cough for 3 weeks but no other symptoms, taking ramipril for hypertension. Ear, throat and chest exams were normal.	Secondary care referral<br>Chest x-ray<br>Chest CT	Antibiotics<br>Oral steroids<br>Anti-tussive medicines<br>Stop ramipril<br>Advise visit to pharmacy to try remedies there<br>Tell her that no action is needed at this stage
	2	3.9	Patient returns after another 3 weeks, saying cough has persisted and now there are streaks of blood in the sputum. No weight loss, but a chest examination reveals some crepitations at the left base. Any tests previously undertaken were normal.		
2 Lung	1	3.6	62-year-old man with COPD, a heavy smoker for over 40 years. He presented with respiratory symptoms.	Secondary care referral<br>Chest x-ray<br>Chest CT	Advise increased use of salbutamol inhaler<br>Antibiotics<br>Oral steroids<br>Antibiotics and add new inhaler- steroid or salmeterol<br>Anti-tussive medicines<br>Advise visit to pharmacy to try remedies there<br>Tell him that no action is needed at this stage
	2	>10.0	Patient returns in 3 weeks, complains of constant ache in left shoulder. Patient attributes pain to persistent cough; he is also producing grey coloured sputum in larger quantities than usual, but no other chest symptoms. No weight loss. On exam he has still a bilateral upper lobe wheeze and some crepitations at the left base. Examination of his shoulder is normal.		
3 Colorectal	1	0.7	43-year-old woman with IBS for more than 10 years, but the IBS has gotten worse recently. She has abdominal pain every day, unchanged bowel habits and no other symptoms. She has no family history of cancer.	Secondary care referral<br>Colonoscopy<br>Abdominal CT	Prescribe medication for IBS<br>Give dietary advice<br>Offer psychological therapies (counselling and CBT)<br>Tell her that no action is needed at this stage
	2	1.2	The patient returns to see you. Her recent blood test has returned a haemoglobin level of 10.5g/dl.		
4 Colorectal	1	0.9	68-year-old man with no relevant medical history. He has experienced loose stools twice a day, most days for over 4 weeks. He has no other symptoms. Examination incl. rectal exam was normal.	Secondary care referral<br>Colonoscopy<br>Abdominal CT	Offer medication e.g. loperamide, antispasmodic, analgesia.<br>Advice on diet<br>Tell him that no action is needed at this stage
	2	1.9	Any tests selected are negative. Patient returns 2 weeks later, describing that the diarrhoea remains much the same, but he now also has intermittent sharp abdominal pain. Abdominal and PR examinations are normal.		
5 Ovarian	1	0.3	53-year-old woman whose last period was 6 months ago. She had experienced abdominal pain for the last 3 weeks. She has had no other symptoms and the same sexual partner for 20 years.	Secondary care referral<br>Abdominal ultrasound<br>Pelvic CT	Prescribe analgesia<br>Prescribe anti-spasmodic<br>Undertake investigations now<br>Tell her that no action is needed at this stage
	2	0.7	All investigations to date have been normal. The patient presents one month later with urinary frequency. She says the abdominal pain is still present but comes less often. Abdominal examination is normal. A urine dipstick for blood, protein, nitrite, white cells and sugar is negative.		
